# Supplementary material for: Management and prognostic prediction of pyogenic liver abscess in a Chinese tertiary hospital: Percutaneous needle aspiration vs catheter drainage
Source: PLoS One. 2024 Dec 16;19(12):e0315371. doi: 10.1371/journal.pone.0315371 (PMC11649130; doi:10.1371/journal.pone.0315371)
Supplement: S1 Appendix — (DOCX) [file pone.0315371.s001.docx]

|  | | | | | |
| --- | --- | --- | --- | --- | --- |
| **Variable** | **OR (95% CI)** | ***P* value** | **Variable** | **OR (95% CI)** | ***P* value** |
| Age | 0.979(0.956-1.003) | 0.080 | Alkaline phosphatase (u/L) | 1.003(1.001-1.004) | 0.008 |
| Gender | 1.116(0.578-2.155) | 0.744 | Bilirubin mg/dl | 1.005(0.999-1.011) | 0.103 |
| Area Rural: Urban | 1.281(0.677-2.424) | 0.447 | Serum creatinine (μmol/L) | 0.997(0.989-1.005) | 0.453 |
| Married | 2.028(0.448-9.179) | 0.358 | Abscess site |  | 0.180 |
| Smoke | 1.283(0.676-2.437) | 0.446 | Left | Reference |  |
| Alcohol intake | 1.411(0.747-2.665) | 0.289 | Right | 0.565(0.144-2.209) |  |
| Liver cirrhosis | 3.360(1.258-8.972) | 0.016 | Both | 1.382(0.467-4.086) |  |
| Fatty liver | 0.468(0.109-2.014) | 0.308 | Abscess size | 0.987(0.872-1.117) | 0.831 |
| Cholelithiasis | 1.155(0.532-2.508) | 0.715 | Intervention of abscess | 2.208(0.998-4.884) | 0.051 |
| Charlson comorbidity index score | 1.322(1.036-1.686) | 0.025 | Organism cultured (pus) | 1.168(0.606-2.249) | 0.643 |
| Previous surgery | 2.511(1.189-5.300) | 0.016 | Organism cultured (blood) | 4.332(2.119-8.857) | < 0.001 |
| Leukocyte (10^9^/L) | 0.983(0.925-1.044) | 0.574 | ICU admission | 7.920(2.044-30.685) | 0.003 |
| C-reactive protein (mg/L) | 1.001(0.997-1.005) | 0.669 | No. of procedure related complications | 0.743(0.392-1.405) | 0.360 |
| Albumin(g/dL) | 0.955(0.899-1.016) | 0.145 | Resolution of fever | 1.047(1.020-1.074) | 0.001 |
| Alanine transaminase (u/L) | 1.002(0.997-1.007) | 0.481 | Hospital stay | 1.033(1.011-1.056) | 0.003 |
| Aspartate transaminase (u/L) | 1.005(1.001-1.009) | 0.014 |  |  |  |

**S1 Appendix. Risk factors for treatment failure in 445 patients with pyogenic liver abscess analyzed by univariate logistic regression**
