# Supplementary material for: Management and prognostic prediction of pyogenic liver abscess in a Chinese tertiary hospital: Percutaneous needle aspiration vs catheter drainage
Source: PLoS One. 2024 Dec 16;19(12):e0315371. doi: 10.1371/journal.pone.0315371 (PMC11649130; doi:10.1371/journal.pone.0315371)
Supplement: Attachment — Submitted filename: comments.docx [file pone.0315371.s002.docx]

1. Why the diameter of the pigtail tube is 8F, and the 8F tube diameter is small, why do not use 10-12F.
2. What are the inclusion and exclusion criteria for the studies? Patients diagnosed with PLA were included? Did the PLA liquefied very good? Have any multilocular liver abscess?
3. Once diagnosed with a bacterial liver abscess, PNA or PCD occurs quickly, and what about patients with low platelets or those taking anticoagulants? Does PNA enhance the bleeding risk
4. Most of the previous studies show that PCD is better than PNA. Is it because the pigtail tube you use has a small diameter? Or is the drain tube placed in a poor position?
5. Does a larger PLA have only one puncture point for PNA treatment?
6. PCD failure needs surgical treatment, why not undertake PNA treatment after PCD treatment failure?
7. The clinical characteristics of patients with PLA treated with PNA and PCD were not compared, could there be a conclusion bias? Could there be a large difference in the average abscess size?
8. What is the use of antibiotics? Is there any significant difference? Will it affect the study results?
9. Previous studies have shown that PNA is mainly used for the treatment of patients with liver abscess with small diameter. If possible, please show the comparative picture. I am interested in the treatment effect of PNA on larger liver abscesses.
10. What are the surgery-related complications, please specify them.
